# SARS-CoV2 Infection and Comorbidity in Inmates: A Study of Central Italy

**DOI:** 10.3390/ijerph20043079

**Published:** 2023-02-09

**Authors:** Emma Altobelli, Francesca Galassi, Marianna Mastrodomenico, Fausto Frabotta, Francesca Marzi, Anna Maria Angelone, Ciro Marziliano

**Affiliations:** 1 Department of Life, Public Health and Environmental Sciences, University of L’Aquila, 67100 L’Aquila, Italy; 2 Public Health Unit, Avezzano-Sulmona-L’Aquila, 67100 L’Aquila, Italy; 3 Department of Information Engineering, Computer Science and Mathematics University of L’Aquila, 67100 L’Aquila, Italy; 4 Statistical Observatory and Indicator Monitoring, University of L’Aquila, 67100 L’Aquila, Italy

**Keywords:** prison, inmate, COVID-19, comorbidity, multiple correspondence analysis

## Abstract

Background and Objective: The presence of multiple chronic diseases is associated with an increase in mortality when related to COVID-19 infection. The aims of our study were: (i) to evaluate the association between the severity of the COVID-19 disease, defined as symptomatic hospitalized in prison or symptomatic hospitalized out of prison, and the presence of one or more comorbidities in two prisons in central Italy: L’Aquila and Sulmona; (ii) to describe the profiles of inmates using multiple correspondence analysis (MCA). Methods: A database was created including age, gender and clinical variables. The database containing anonymized data was password-protected. The Kruskal–Wallis test was used to evaluate a possible association between diseases and the severity of COVID-19 stratified by age groups. We used MCA to describe a possible characteristic profile of inmates. Results: Our results show that in the 25–50-year-old age group (COVID-19-negative) in the L’Aquila prison, 19/62 (30.65%) were without comorbidity, 17/62 (27.42%) had 1–2 comorbidities and only 3.23% had >2 diseases. It is interesting to note that in the elderly group, the frequency of 1–2 or >2 pathologies was higher than in the younger group, and only 3/51 (5.88%) inmates did not have comorbidities and were COVID-19 negative (*p* = 0.008). The MCA identified the following profiles: the prison of L’Aquila showed a group of women over 60 with diabetes, cardiovascular and orthopedic problems, and hospitalized for COVID-19; the Sulmona prison presented a group of males over 60 with diabetes, cardiovascular, respiratory, urological, gastrointestinal and orthopedic problems, and hospitalized or symptomatic due to COVID-19. Conclusions: our study has demonstrated and confirmed that advanced age and the presence of concomitant pathologies have played a significant role in the severity of the disease: symptomatic hospitalized in the prison; symptomatic hospitalized out of the prison.

## 1. Introduction

It is known that in prisons, the risk to the health of prisoners is greater if there is overcrowding and if they present comorbidities [1,2,3].

Italy was among the first countries in Europe to be affected by the COVID-19 pandemic [4,5,6,7]. This required the definition and implementation, in the shortest possible time, of management and prevention protocols, both for the Italian and prison populations. By definition, prisons represent closed, highly complex community structures, whose health services in Italy are managed by the Ministry of Health [8].

Italy is in third place in Europe in terms of prison population density, with an occupancy rate of 115% [9] and inadequate infrastructure in general. As of 22 November 2022, there are 55,734 inmates in 189 institutes, compared with a regulatory capacity of 50,942 places [10]. 

It is known that the prison population does not, on average, enjoy the same state of health as the rest of the free population [11]. Several studies have shown that prisons, also due to socio-economic and health determinants [12,13], are characterized by a higher prevalence of chronic diseases and an increased risk of contagion from infectious diseases, including COVID-19, compared to non-confined contexts [14]. The presence of a pathological condition, even if not serious, was found in 67.5% of the total number of prisoners present in 57 prisons in Italy, and an average of 2.2 diseases was reported for each prisoner [15,16].

Furthermore, a high number and the presence of specific comorbidities affect the immune response in patients who test positive for COVID-19 infection [17], presenting an increased risk of in-hospital mortality following infection. The presence of multiple pre-existing chronic diseases is significantly associated with an increase in the probability of mortality compared to the absence of comorbidities [18]. Age is also closely related to progression and a poor prognosis in patients with COVID-19 infection [19,20].

The aims of our study were: (i) to evaluate the association between the severity of the COVID-19 disease defined as symptomatic hospitalized in prison or symptomatic hospitalized out of prison, and the presence of one or more comorbidities in two prisons in central Italy: L’Aquila and Sulmona; (ii) to describe the profiles of inmates using multiple correspondence analysis.

## 2. Materials and Methods

A database was created including data from medical records of both prisons relating to the period under study: November 2020–August 2022.

The authorization to process the data was obtained on 3 August 2021 (protocol number 0169071/21). Furthermore, the study protocol was approved by the Internal Review Board of the University of L’Aquila (protocol number 41795) on 5 April 2022. In using the data, the privacy and confidentiality of the inmates were guaranteed. For this purpose, technical and organizational measures were put in place in compliance with the principle of data minimization and anonymization. No ethnic or other identification information was codified. The database containing anonymized data was password-protected. 

L’Aquila houses prisoners according to the law of 10 October 1986, n. 663, art. 41 bis, which identifies the so-called “hard prison” in Italy. It is a form of restrictive detention intended for individuals who commit offenses related to organized crime. The 41 bis has 2 main characteristics: the limitation of the outdoor activity of the detainees to a maximum of 2 h and a maximum number of 3 inmates with whom an inmate can socialize. The 41 bis is applied for very long periods, even 20/30 years, a real form of isolation.

Sulmona houses prisoners of the “high security circuit” known as 1 and 3. Furthermore, it also houses collaborators of justice (art. 21 of the Penitentiary Regulations). Prison circuit 1 is dedicated to prisoners who have committed Mafia crimes; circuit 3 houses inmates who have committed crimes related to drug dealing.

The variables available regarding inmates were: age at the time of enrollment in the cross-sectional study, gender, COVID-19-positive and -negative cases, the severity of the disease: symptomatic hospitalized in the prison or symptomatic hospitalized out of the prison and the presence or absence of comorbidities classified according to the number of diseases (absence, 1–2 and >2 comorbidities). The groups of comorbidities analyzed were: respiratory, cardiovascular, urological, orthopedic, gastroenteric diseases, type 2 diabetes, renal chronic insufficient, hematologic diseases, cancers, immunodeficiency and HIV. Age was divided into 3 classes: 25–50, 51–60 and >60 years old.

The following tests were used to diagnose SARS-CoV2 infection: Hologic, COPAN Italia S.p.A. (molecular test) and a SARS-CoV2 Antigen Rapid test kit. 

For the L’Aquila prison alone, stratification by sex was carried out to evaluate the distribution of frequency, because Sulmona contains only males. Subsequently, we used multiple correspondence analysis (MCA) to describe a possible characteristic profile of inmates. We have included the variables described above in the MCA statistical models. The database was managed using Microsoft Excel software. The mean age was calculated as mean ± standard deviation (SD).

The Kruskal–Wallis test was used to evaluate a possible association between the number of diseases and the severity of COVID-19, stratified by the age groups considered. Multiple correspondence analyses were applied using the Facto Mine R library of statistical software R (Version R 4.2.2, R Foundation for Statistical Computing, Vienna, Austria).

## 3. Results

The distribution of the prison population in the two penitentiary institutes of the Abruzzo region is shown below. The population of the Sulmona prison is made up of 424 males, with mean age and SD of 52.7 ± 11.18. The L’Aquila prison is made up of a population of 150 males (92.02%) and 13 females (7.98%), with a mean age and SD of 51.81 ± 11.07 for males and 63.31 ± 8.66 for females. Moreover, L’Aquila showed a mean age for males of 53.19 ± 9.07 vs. 50.61 ± 11.07, respectively, positive vs. negative (*p* = 0.15), and for females, 61.80 ± 9.05 vs. 63.31 ± 8.66 (*p* = 0.27), respectively, positive vs. negative; Sulmona showed the mean age was for males 56.80 ± 11.29 vs. 51.11 ± 10.75, respectively, positive vs. negative (*p* = 0.001).

In L’Aquila, 49.1% of the prisoners tested were positive for COVID-19, of which 38.7% belonged to the age group between 25 and 50 years old, 66.0% to the group between 51 and 60 years and 45.1% belonged to the highest group of >60 years. 

In Sulmona, the percentage of positives was 28.3%, 17.6% of them were in the 25–50-year-old age group, 31.7% in the 51–60 age group and 43.5% in those over 60 years of age.

Table 1 shows the frequencies of the variables analyzed relating to the inmates of both prisons of L’Aquila and Sulmona. Our results show that the most common comorbidities in inmates of L’Aquila are cardiovascular and orthopedic diseases, whereas for Sulmona, they are orthopedic and vascular diseases.

Table 2 shows the following stratified frequency distributions for the prisons of L’Aquila and Sulmona and for age groups according to the number of diseases and their severity due to COVID-19.

Subsequently, the association between the severity of COVID-19 disease and the presence of comorbidities, stratified by age group, was evaluated for the inmates of both prisons (Table 3).

Regarding the 25–50-year-old age group (COVID-19-negative) in L’Aquila, 19/62 (30.65%) were without comorbidity, 17/62 (27.42%) had 1–2 comorbidities and only 3.23% had >2 diseases. In this age group, only 8% were hospitalized out of the prison despite the presence of >2 pathologies (*p* = 0.0001). On the other hand, in the 51–60y age group, there was no statistically significant association between disease severity and the number of comorbidities (*p* = 0.17).

Instead, it is interesting to note that in the elderly group, the frequency of 1–2 or >2 pathologies was higher than in the younger group, and only 3/51 (5.88%) of inmates did not have comorbidities and were COVID-19 negative (*p* = 0.008). It is important to underline that only 1/50 (2.0%) of the 51–60y group with >2 diseases needed an external hospitalization. In total, for both prisons, external hospitalizations were 11. Analyzing the results relating to the prisoners in Sulmona, no statistically significant differences were observed between the severity of the COVID-19 disease and the presence of comorbidities in any age group, respectively, *p* = 0.73, *p* = 0.33 and *p* = 0.29/163 (11.04%) (Table 3). 

Therefore, both in L’Aquila and in Sulmona, in the group of restricted subjects with >2 concomitant pathologies, there was a rising trend of infection due to an increase in age. None of the inmates of L’Aquila and Sulmona were admitted to intensive care.

**Figure 1:**
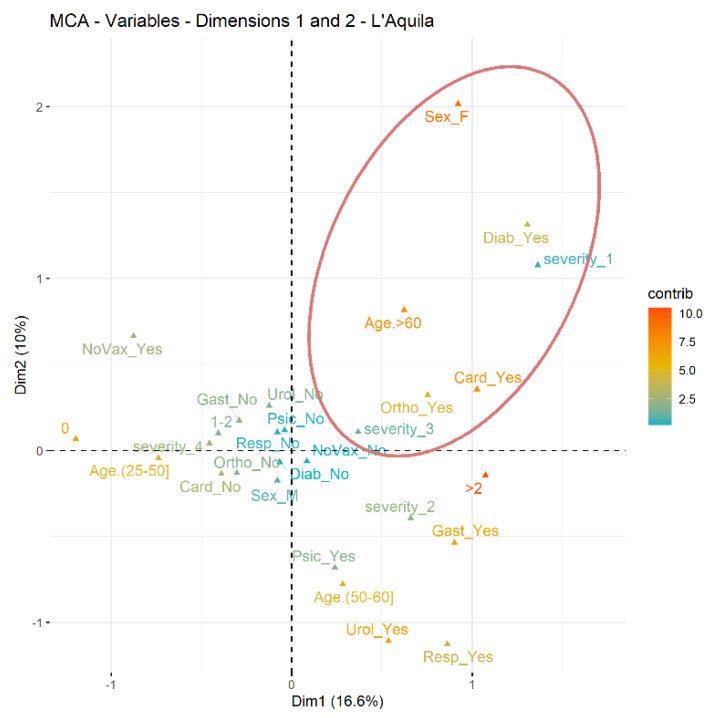
L’Aquila Prison.

**Figure 2:**
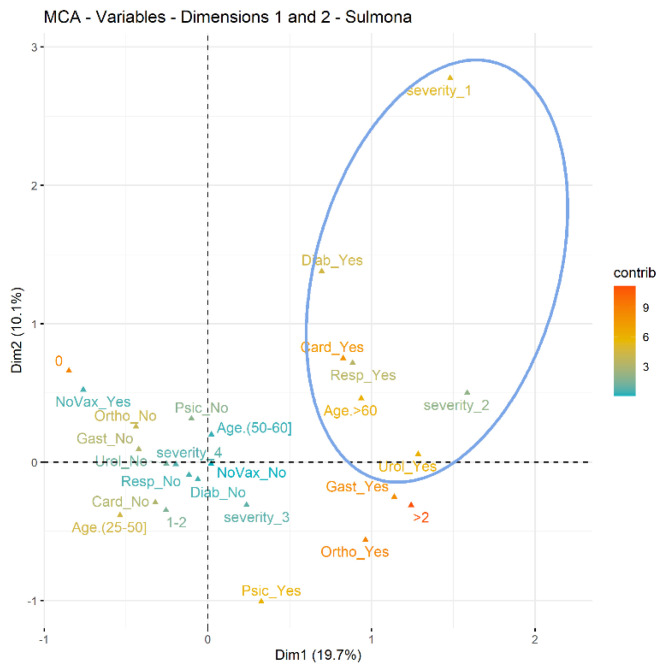
Sulmona Prison.

Finally, we conducted the analysis of multiple correspondences to identify groups of prisoners who developed the most serious forms of COVID-19: the prison of L’Aquila showed a group made up of women, aged over 60, with diabetes, with cardiovascular and orthopedic problems and hospitalized for COVID-19; the Sulmona prison presented a group of inmates aged over 60 with diabetes, with cardiovascular, respiratory, urological, gastrointestinal and orthopedic problems and hospitalized for COVID-19 (Figure 1 and Figure 2).

## 4. Discussion

The SARS-CoV-2 health emergency that affected the whole world inevitably had repercussions on the prison situation. The perception that penitentiary facilities are isolated and separated environments from the rest of society—and, therefore, more protected from the risk of contagion—has proved to be illusory and wrong, colliding with reality and everyday prison life [21].

The restricted population is considered “fragile”, not only due to some determinants such as the condition of the deprivation of personal freedom, overcrowding and low sanitation standards but also because it has a greater burden of chronic, infectious diseases at an advanced age which places it at a higher risk of infection [22,23,24].

Our study has demonstrated and confirmed that advanced age and the presence of concomitant pathologies have played a significant role in the severity of the disease: symptomatic hospitalized in prison and symptomatic hospitalized out of prison. We therefore identified characteristic profiles of inmates, determined through the multiple correspondence analysis, confirming in both institutions at an age greater than 60 the presence of cardiovascular diseases, diabetes mellitus and orthopedic disorders. In Sulmona, the profile is also characterized by pathologies affecting the respiratory and urological systems. The conditions of multimorbidity, commonly present among prisoners, appear more often with an early onset and with greater severity than in the general population [25] since the stress inmates are subjected to can favor the onset of pathologies [20].

In prisoners, the stress due to the rehabilitation imposed by the prison environment leads to an acceleration of aging, to the point that some 50-year-olds present psycho-physical conditions more typical of subjects over 65 [26].

The odds of a 50-year-old prisoner becoming ill would be the same as for a 60-year-old outside of the prison [27].

In Italy, the health of the prison population was the subject of a multi-center study financed by the Ministry of Health, which involved over 15,000 prisoners present in 57 structures [15]. This study showed that 67.5% of people detained in Italy are affected by at least one disease, and it is important to underline that our results are in line with the national data, 70.4%.

Furthermore, it is important to highlight that in the prison environment, all the main risk factors for cardiovascular diseases are found: cigarette smoking, a diet rich in fats, a sedentary lifestyle, overweight and obesity [28].

A more recent study [20], carried out on the Italian prison population, demonstrated that heart disease had the highest percentage in the sample of elderly prisoners. 

S.A. Kinner et al. state that in prisoners, as in the general population, the number of comorbidities increases with age [22,29].

Regarding the SARS-CoV2 infection, the international literature has shown a more severe form among elderly people and in those with comorbidities [6,26].

These results are largely confirmed in our study. In fact, it has emerged that there is a statistically significant association between the number of comorbidities and the severity of the COVID-19 disease in reference to different age groups. In the 25–50-year-old age group, 30.65% were negative and without comorbidities. On the contrary, only 5.88% of older prisoners did not have comorbidities and were COVID-19-negative.

In particular, in L’Aquila and Sulmona, the inmates who required hospitalization in or out of the same institution were over 60 years of age, with one or more comorbidities. However, the infections did not cause intensive care admissions or deaths.

A limitation of our study is that it was not possible to compare our comorbidity data with those of the Italian population, with those of the 12 Italian prisons (41 bis) or with those of maximum-security institutions because they are not available in open source. Another limitation regards the problem of overcrowding which was not possible to evaluate because those two correctional settings were very different with respect to the rest of Italy for these types of criminal provisions. In fact, L’Aquila is a form of detention with a 2 h outdoor maximum limitation for the detainees and a maximum number of three inmates that an inmate can socialize with. Sulmona is a “high security circuit” that consists of limitations aimed at reducing the frequency of contact with the outside world. It is therefore a preventive tool that aims to “isolate” the person from the rest of the criminal organization. Therefore, it is not possible to hypothesize which measures to adopt for the health protection of inmates.

## 5. Conclusions

Our study has demonstrated and confirmed that advanced age and the presence of concomitant pathologies have played a significant role in the severity of the disease: symptomatic hospitalized in prison and symptomatic hospitalized out of prison. Prison health care plays a fundamental role in identifying the health needs of inmates, and in detecting acute and chronic conditions of suffering, even if the assistance presents considerable difficulties due to constraints linked to the condition imposed by the limitation of freedom.

In conclusion, our study confirms that the implementation of the continuous monitoring of the health status of prisoners is crucial.

## Figures and Tables

**Table 1:** Distribution of comorbidity.

	L’Aquila Prison		Sulmona Prison	
Variables	Present No. (%)	Absent No. (%)	Present No. (%)	Absent No. (%)
Respiratory	14 (8.59)	149 (91.41)	49 (11.56)	375 (88.44)
Cardiovascular	45 (27.61)	118 (72.39)	119 (28.07)	305 (71.93)
Type 2 DM	8 (4.91)	155 (95.09)	35 (8.25)	389 (91.75)
Renal Chronic Insufficient	0 (0.00)	163 (100.00)	2 (0.47)	422 (99.53)
Hematologic disease	8 (4.91)	155 (95.09)	10 (2.36)	414 (97.64)
Cancers	10 (6.13)	153 (93.87)	20 (4.72)	404 (95.28)
Immunodeficiency	1 (0.61)	162 (99.39)	1 (0.24)	423 (99.76)
HIV	2 (1.23)	161 (98.77)	2 (0.47)	422 (99.53)
Urological disease	31 (19.02)	132 (80.98)	70 (16.51)	354 (83.49)
Orthopedic disease	47 (28.83)	116 (71.17)	133 (31.37)	291 (68.63)
Gastroenteric disease	40 (24.54)	123 (75.46)	115 (27.12)	309 (72.88)

**Table 2:** Distribution of comorbidity frequency of inmates according to gender: L’Aquila.

Variables	Females		Males	
	Present No. (%)	Absent No. (%)	Present No. (%)	Absent No. (%)
Respiratory	1 (0.08)	12 (0.92)	13 (0.09)	137 (0.91)
Cardiovascular	6 (0.46)	7 (0.54)	39 (0.26)	111 (0.74)
Type 2 DM	2 (0.15)	11 (0.85)	6 (0.04)	144 (0.96)
Renal Chronic Insufficient	0 (0.00)	13 (1.00)	0 (0.00)	150 (1.00)
Hematologic disease	1 (0.08)	12 (0.92)	7 (0.05)	143 (0.95)
Cancers	1 (0.08)	12 (0.92)	9 (0.06)	141 (0.94)
Immunodeficiency	0 (0.00)	13 (1.00)	1 (0.01)	149 (0.99)
HIV	0 (0.00)	13 (1.00)	2 (0.01)	148 (0.99)
Urological disease	0 (0.00)	13 (1.00)	31 (0.21)	119 (0.79)
Orthopedic disease	6 (0.46)	7 (0.54)	41 (0.27)	109 (0.73)
Gastroenteric disease	3 (0.23)	10 (0.77)	37 (0.25)	113 (0.75)

**Table 3:** Association between the number of comorbidities and the severity of COVID-19 disease stratified by age group.

Age-group	25–50 (Total 62)					51–60 (Total 50)					>60 (Total 51)				
Severity	Hosp out	Hosp in	Asymp	Neg	*p*	Hosp out	Hosp in	Asymp	Neg	*p*	Hosp out	Hosp in	Asymp	Neg	*p*
No. of co.															
L’Aquila Prison															
0 No %	0 0.00	0 0.00	2 3.23	19 30.65	0.0001	0 0.00	1 2.00	5 10.00	2 4.00	0.17	0 0.00	0 0.00	0 0.00	3 5.88	0.008
*1–2* No %	0 0.00	4 6.45	9 14.52	17 27.42		0 0.00	1 2.00	10 20.00	8 16.00		0 0.00	2 3.92	3 5.88	15 29.41	
*>2* No %	0 0.00	5 8.06	4 6.45	2 3.23		1 2.00	7 14.00	8 16.00	7 14.00		0 0.00	6 11.76	12 23.53	10 19.61	
Sulmona Prison															
Age-group	25–50 (total 193)					51–60 (total 123)					>60 (total 108)				
Severity	Hosp out	Hosp in	Asymp	Neg	*p*	Hosp out	Hosp in	Asymp	Neg	*p*	Hosp out	Hosp in	Asymp	Neg	*p*
No. of co.															
0 No %	0 0.00	1 0.52	13 6.74	68 35.23	0.73	1 0.81	0 0.00	10 8.13	28 22.76	0.33	1 0.93	0 0.00	5 4.63	15 13.89	0.29
*1–2* No %	0 0.00	0 0.00	11 5.70	67 34.72		0 0.00	2 1.63	9 7.32	35 28.46		2 1.85	2 1.85	8 7.41	17 15.74	
*>2* No %	0 0.00	1 0.52	8 4.15	24 12.44		2 1.63	2 1.63	13 10.57	21 17.07		4 3.70	6 5.56	19 17.59	29 26.85	

Legend: Co: comorbidity; Hosp in: symptomatic hospitalized in prison; Hosp out: symptomatic hospitalized out prison; Asymp: asymptomatic; Neg: negatives.

## Data Availability

Not applicable.
